# Difficulties in the diagnosis and treatment of rare diseases according to the perceptions of patients, relatives and health care professionals

**DOI:** 10.6061/clinics/2018/e68

**Published:** 2018-03-24

**Authors:** Marcos Thomazin Lopes, Vera Hermina Koch, Vicente Sarrubbi-Junior, Paulo Rogério Gallo, Magda Carneiro-Sampaio

**Affiliations:** I Pediatria, Instituto da Crianca, Hospital das Clinicas HCFMUSP, Faculdade de Medicina, Universidade de Sao Paulo, Sao Paulo, SP, BR; II Saude Materno Infantil, Faculdade de Saude Publica, Universidade de Sao Paulo, Sao Paulo, SP, BR

**Keywords:** Diagnosis, Therapy, Rare Diseases, Research, Qualitative Study, Vulnerability

## Abstract

**OBJECTIVES:**

The aim of this study is to present a survey of vulnerabilities and to suggest approaches for the treatment of rare diseases according to the perceptions of a group of affected individuals, patient association representatives and health care professionals.

**METHODS:**

The focus group technique was used in interviews with patients and primary caregivers, patient support groups/non-governmental organizations, primary health care professionals and physician specialists.

**RESULTS:**

The transcript analysis focused on thematic units, which were tailored to each group and allowed comparisons in search of concordant views. Unanimity was observed in relation to the physical, emotional and social damage to the life standards of the affected individuals and their families as a result of illness. The Brazilian health system was unanimously classified as inadequate to respond to the needs of patients with rare diseases, and this inadequacy led to unpleasant experiences, such as the seemingly endless referrals among health services to reach a final diagnosis and develop a treatment plan.

**CONCLUSIONS:**

The complex set of health system requirements necessary to support the care of patients with rare diseases represents an obstacle to successfully meeting the needs of patients and their families. Therefore, it is important to develop specific public policies to create referral services, guarantee access to appropriate therapeutic modalities and incorporate technologies that promote research for developing new, affordable therapies.

## INTRODUCTION

According to the World Health Organization, a disease is considered rare when it affects 1.3 in 2000 persons. There are approximately 7,000 known rare diseases (RDs), 80% of which are genetic in origin; the remaining diseases may be caused by environmental, infectious or immunological factors 1-4.

RDs are known for their unique, complex characteristics, which may lead to chronic clinical deterioration and gradual interference with social, physical and psychological aspects of the patient’s daily life, with a direct negative impact on the overall wellbeing of the nuclear family 1,3.

The public health system has been the primary source of RD diagnosis and treatment. However, the lack of structured health policies and regional referral centers forces patients and caregivers to face an endless pilgrimage to successive health centers in search of a final diagnosis and proper treatment 2.

Considering the need to individualize and prioritize RD diagnosis and treatment, the present study aimed to evaluate vulnerabilities and suggest approaches for RD diagnosis and treatment in Brazil based on the perceptions of those involved in the process: patients, caregivers, patient support groups, non-governmental organizations and primary and tertiary care professionals 5,6.

## METHOD

This cross-sectional qualitative study used non-random sampling. The study included 27 participants divided into four groups: patients and primary caregivers (PC; G1); patient support groups and non-governmental organizations (G2); primary care health professionals (G3); and tertiary care physician specialists (G4), as described in Table 1. After the study objectives were presented, all the participants signed an informed consent form.

The researchers involved in the study served as moderators and reporters in each group to ensure procedural homogeneity. A script containing guidelines was presented to each group before the session was initiated 7-9.

The reports were recorded and transcribed in full and served as a basis for the thematic and categorical content analysis 10,11. The thematic units were coded and processed with NVivo 10 software, which allowed us to map their distribution among the different study groups 12,13. For better reliability, data triangulation was used to achieve the highest degree of convergence among the researchers' perceptions.

The procedures adopted in this study followed the ethical criteria for research on humans, as written in resolution number 466/2012 of the National Health Council. This study was approved by the Ethics Committee for Review of Research Projects (Comitê de Ética para Análise de Projetos de Pesquisa - CAPPesq) of Hospital das Clínicas of the FMUSP (process 268.417, May 8, 2013) 14.

## RESULTS AND DISCUSSION

The group approach promoted a high level of interaction among the participants, which enhanced the exchange of ideas regarding the multiple encountered difficulties related to diagnosis and access to treatment modalities. Intense discussions and vivid reports were equally present in the four study groups. The analysis concentrated on thematic units in terms of the group in which they occurred, which allowed the detection of the participants’ consonant opinions regardless of the stratum to which they belonged (Table 2).

As chronic diseases, RDs present an extended course that negatively affects several functional features and impact multiple aspects of the patient’s life, including the frequent impairment of inclusion in social activities. These consequences tend to involve the entire family, particularly the inner circle of relatives, creating high levels of stress and the possible disruption of family ties and physical problems, as described by Goble (2004) and in fragments of the “speeches” recorded in the present study during the interactive group activity 1,15-19. These fragments are presented below in *italic print*.

You lose all your friends, your whole family; your doorbell and phone do not ring anymore because whenever you call your relatives, they already think you are going to ask for something, and when you do, they label you an opportunist. “You are willing to take advantage of your child’s disease; you are using the disease to travel and obtain financial advantages.” (G1)

My daughter started to show symptoms of lung disease at an early age. She failed her last year of senior high school because she constantly felt sick, and because of this, she was accused of being lazy, but in fact, she was tired from not being able to breathe. (G1)

I lost my job due to prejudice and was reinstated by court decision. Such is a sick person’s life in Brazil: on top of the burden of disease, we carry a very strong stigma, which deeply affects us emotionally. (G1)

The possibility of experiencing social stigma on top of physical and psychological pain was present in the narratives of the patients and their caregivers. Part of this suffering could be alleviated with a prompt and correct diagnosis, which would facilitate the development of coping strategies and the search for supportive treatment.

However, the present study shows that during the search for a diagnosis, an individual with an RD is exposed to situations that increase their feelings of fragility and helplessness, as described below.

The greatest difficulty is the establishment of an accurate diagnosis. So frequently, these patients circulate from one health facility to another until a given disease is suspected or identified and they are finally referred to appropriate specialists. (G2)

Therefore, the person is already fragile, particularly nowadays. It is more understandable for a person of my age, but it is very difficult for a younger person who stands there like a guinea pig. “They” brought a jug with hot water, dipped my daughter’s hand in it and then put her hand in a bowl of ice. You can imagine the pain she felt, as she has a skin disease. She passed out from the pain. She had to suffer all this in the presence of medical students for them to identify her disease. From that day on, I have never taken my daughter back to a university hospital. I saw her fainting from pain. “Ah, let’s see how that hand looks”. Everyone holding a camera; one brings hot water and puts her hand in it, which starts to become cyanotic, and then puts it on the ice. The child could not stand it. My pain was even stronger for having allowed such a procedure. Then, she told me: “Mom, I don’t want this anymore. Let me die.” (G1)

Although current advances in the medical field have facilitated the diagnosis of various diseases, the scientific and technological arsenal is ineffective if it is not made available to health professionals during academic training or through continued medical education. RD recognition is highly dependent on the effective transfer of updated scientific information to health professionals 3,4,16,18-21. This reality is described below.

Many professionals have never heard of (some types of RD). Doctors, OB-GYN specialists, nurses, pediatricians, neonatal medicine specialists... have never seen it, and when they face it, their clinical interpretation of the clinical findings may lead them in the wrong direction (G3).

The patient’s journey does not end with the diagnosis of the disease; another significant obstacle faces health personnel and relatives after diagnosis: the challenge of searching for adequate treatment, which, at least in Brazil, is far from straightforward. The Brazilian public health system has been unable to meet the needs of RD patients who, due to the multiplicity of these diseases and their rarity, are frequently unable to organize a strong and representative support group, as described below 1,20.

The challenges are huge, and we realize it. There is a waiting list to get an appointment with a specialist that sometimes, depending on the specialty, is longer than one year. Sometimes, the patient dies before the scheduled clinic appointment because he was not rated as a priority (G2).

Primary care health professionals and physician specialists also experience great discomfort regarding RD patients because the reference and counter-reference system is slow and ineffective and presents another obstacle on the journey to treatment, as described below.

There are difficulties in referring the patient to a specialized center. The information regarding where to refer patients with specific diseases is not organized. Depending on the disease, the patient will need additional care provided by non-medical health professionals, and these may not always be available – and many times are out of reach. (G3)

The desire to have a network (interconnected health services) is very strong, but it is not available. There is no organized management mechanism; the patient and his family go back and forth with the reference request document for a long time before they reach the right place. (G2)

From a clinical point of view, it is fundamental that we are able to conduct a presumptive diagnosis for the patients arriving in critical condition. Exams based on simple biochemical methodology are available, but whenever a more accurate diagnosis is necessary, such as an enzymatic or genetic determination, these techniques are generally unavailable, and the final diagnosis is delayed. It is possible to send the biological samples to another state or to another country, but it may take one or two months to get the results back, and the child may die before the results arrive. Another issue is financial; the hospital cannot always afford to send the biological samples to another center, and therefore, we may find ourselves very alone, trying to perform miracles or find a friend who may help us for free in order to reach a presumptive diagnosis. (G4)

The global financial and economic context has promoted the adoption of cost-saving measures, which inevitably has taken a toll on health systems. Therefore, the legal battle for access to therapies that the health system does not consider a priority has been the most effective strategy for many patients once conventional alternatives have been exhausted.

This practice, however, has been widely debated in the legal environment, where it has triggered considerable controversy because while public managers aim to allocate resources to promote universal access to health care, the judicial system tries to ensure the rights and meet the needs of patients with RDs, as described by Andrade (2008) 16.

Although judicialization is the fastest and the most reliable method for legitimizing access to individual therapeutic assistance, its indiscriminate use deepens the perversity of the health system because it favors certain groups over others. However, some of the actions taken could be avoided with investments in the development of technologies aimed at differentiated consumers.

Regarding medications for RDs or “orphan drugs”, there is a lack of incentive for the national pharmaceutical industry to conduct research and testing related to the manufacture of these medications. Consequently, these drugs must be imported, which leads to greater public costs 4-6,22-26.

In fact, it (the orphan drug) has not been released by ANVISA (the National Health Surveillance Agency), but we can obtain it through existing laws that regulate orphan drugs. In cases in which there is only one drug for a disease on a worldwide basis, Brazilian law will then release it provided it is approved in Europe or in the United States. By law, ANVISA is obligated to release medicine for import, and we have already obtained some as a result of this fact. (G3)

Although the Unified Health System [Sistema Único de Saúde - SUS] has several flaws in its main programs for the general population, the people interviewed in the present research presented innumerable suggestions for improvements to meet the needs of minority groups.

The most commonly cited suggestions for optimizing the medical care provided to RD patients were to increase investment in the training and qualification of health professionals and to create a model of care that includes an efficient communication flow between multi-professional teams of primary care and referral/specialized professionals that are responsible for diagnosis, treatment and providing psychological support to patients and their families, as described below.

It is my opinion that a course should be included in the university academic curriculum to introduce the RD topic, as there currently is none. (G1)

Another important aspect is to qualify and train professionals so they can have an accurate perception (of RDs), to conduct home visits as early as possible, and to work in conjunction with the community and community health agents because they are the connecting point between the community and health professionals. (G2)

It is necessary to have a multidisciplinary team to take care of the child (with an RD) starting at her/his birth. Additionally, it is necessary to have someone to support the desperate family. That way, the treatment will reach not only the child but also the family as a whole. (G3)

Reference centers for research, diagnosis, treatment and the dissemination of information on RDs exist in countries such as Portugal, Belgium, Italy and the United Kingdom. In our society, the existence of centers for the most prevalent diseases and for specific groups provides a glimpse into the possibility of creating centers for people affected by an RD. This system has been suggested as an alternative to avoid the burnout of professionals, caregivers and patients, as described below 1,3,4.

Considering that everyone needs a multidisciplinary team, an institute for this purpose should be created since the prevalence of different RDs is high. (G1)

Considering that both women’s and children’s institutes are already in place, I understand a specific institute for RDs should be created. (G1)

We (tertiary care centers) function at primary, secondary and tertiary levels. We receive patients from all over the country who are erroneously diagnosed and referred for the care of a certain disease because the primary doctor was not able to determine a proper clinical diagnosis and sent the patient to our attention for a solution. This is a waste of time and money, but it is much easier for the state of origin to pay for the patient’s trip to come to our hospital (Hospital das Clínicas in São Paulo City). (G4)

Even if there is no health system that can serve all who need it equally, this should not be an excuse for individuals with responsibilities in these systems. To that effect, recent national initiatives such as Ordinance 199, which was passed by the Ministry of Health on January 30, 2014, and the Brazilian Law of Inclusion, which has been in effect since July 6, 2015, are attempts to rethink inclusion policies and to direct attention toward people affected by some type of RD 27,28.

## CONCLUSION

The complexity of RDs presents obstacles and severe stress factors for patients, their families, and health professionals. To address these obstacles, the present study underscores the need to reduce the time between RD diagnosis and treatment initiation.

The most significant challenge in the Brazilian environment lies in the urgent need to disseminate awareness about RDs in the academic environment and to create public policies that ensure access to orphan drugs and rehabilitation, such as those available in many developed countries. Furthermore, it is common sense that additional needs of RD patients could be met if appropriate technologies were directed toward research aimed at developing new, more accessible and affordable therapies.

## AUTHOR CONTRIBUTIONS

Lopes MT was responsible for the moderation of the focus group, transcription and analysis of recordings, analysis of results and manuscript writing. Koch VH was responsible for the moderation of the focus group, analysis of the results and manuscript writing. Sarrubbi-Junior V was responsible for the analysis of recordings and manuscript writing. Gallo PR was responsible for the analysis of recordings. Carneiro-Sampaio M was responsible for the study coordination.

## Figures and Tables

**Table 1:** Distribution of the participants in the focus groups according to homogeneity criteria.

Group	Composition	Number of participants	Guiding questions	Duration
1	Patients and primary caregivers	07	• Diagnosis • Access to treatment • Rehabilitation therapy	92 min
2	Non-governmental associations and organizations	09	• Diagnosis • Referrals and access to treatment • Community resources and child and family support	87 min
3	Primary care health professionals	04	• Dynamics of caring for patients with RDs • Primary health care and patient care	58 min
4	Physician specialists	07	• Characterization of the activities of doctors • How to qualify experiences in the care of patients with RDs	65 min

**Table 2:** Distribution of thematic units among the groups.

Thematic units	Groups in which the theme emerged
Physical, emotional and social repercussions experienced by patients and caregivers	1 and 3
History of “pilgrimage”	1, 2 and 3
Delays in examination, treatment and medicine	1, 2, 3 and 4
Difficulties encountered by doctors in the diagnosis of patients	1, 2, 3 and 4
Difficulties encountered by doctors in providing treatment	2, 3 and 4
Judicialization as a way to gain access to treatment	1 and 3
Training and qualification of health professionals	1, 2, 3 and 4
Creation of a referral center and a referral and counter-referral system for the dissemination of information on RDs	1, 2, 3 and 4

Groups 1: Patients and primary caregivers; 2: Primary health care professionals; 3: Non-governmental associations and organizations; 4: Physician specialists.
